# Lower Limb Biomechanical Outcomes Following Endoprosthetic Reconstruction for Distal Femur and Proximal Tibia Bone Tumors: A Systematic Review and Meta-Analysis

**DOI:** 10.3390/bioengineering12121310

**Published:** 2025-11-28

**Authors:** Yidan Gao, Bojian Yang, Quan Zhang, Guangming Hu, Yancheng Liu, Kuan Zhang, Jun Sun, Songhua Yan

**Affiliations:** 1 School of Biomedical Engineering, Capital Medical University, Beijing 100069, China; gaoyidan@mail.ccmu.edu.cn (Y.G.);; 2 Department of Bone and Soft Tissue Tumor Oncology, Tianjin Hospital, Tianjin 300211, China; 3 Department of Neurology, Xuanwu Hospital, Capital Medical University, Beijing 100053, China; 4 National Center for Neurological Disorders, Beijing 100053, China

**Keywords:** biomechanics, bone neoplasms, endoprosthetic reconstruction, knee arthroplasty

## Abstract

Given the higher incidence of bone tumors in younger populations, achieving post-operative biomechanical stability is critical to maintaining long-term mobility. The objective of this study was to evaluate the biomechanical impact of endoprosthetic reconstruction in patients with distal femur and proximal tibia bone tumors in comparison with a control group. The Embase, Scopus, PubMed, and Cochrane databases were systematically searched until January 2025, according to PRISMA guidelines. Heterogeneity was assessed via Cochran’s Q statistic and quantified using the I^2^ statistic. A total of 23 studies with 692 participants were included, providing data on gait, knee muscle strength, energy expenditure during walking, physical activity level, balance, and joint position sense. Patients demonstrated significant reductions in gait velocity, cadence, and stride length relative to healthy controls, with abnormalities also observed in ground reaction forces, joint internal moments, and joint power. Additionally, both knee flexion strength and extension strength decreased markedly. This study highlights that endoprosthetic reconstruction substantially altered the biomechanical characteristics of the lower limb in patients with distal femur and proximal tibia tumors. These findings have shown the need for further refinement of surgical techniques, rehabilitation strategies, and follow-up programming.

## 1. Introduction

Primary malignant bone tumors are a rare form of cancer, most commonly diagnosed in individuals under 20 years of age [1,2], with 3770 new cases and 2190 deaths anticipated in the United States in 2025, representing 0.2% of all cancer diagnoses and 0.4% of cancer-related mortality [1]. The knee joint is one of the most commonly affected anatomical sites [3,4]. Historically, conventional treatment approaches for these tumors comprised amputation and limb salvage surgery (LSS). Contemporary comparative analyses have demonstrated that LSS correlates with reduced metastatic incidence and superior 5-year survival rates relative to amputation [5]. Consequently, 85% of cases are currently managed through LSS, establishing it as the standard of care [6]. Advances in oncologic surgical techniques and neoadjuvant therapies have positioned endoprosthetic reconstruction as the dominant therapeutic strategy, superseding amputation as the previous standard intervention [6,7]. As a result, the overall survival of patients is above 60% [8,9,10], and the 5-year survival rate of patients under 64 years is 70% [2].

However, previous studies have shown that the quality of life of bone tumor survivors is reduced, and the prevalence of physical illnesses and psychological problems is higher compared with matched control groups [11,12]. Reduced physical mobility has been documented in patients with lower extremity osteosarcoma relative to other sarcoma subtypes [13,14]. Postoperatively, patients are at risk of developing abnormal gait kinematics, characterized by knee hyperextension, joint stiffness, and restricted range of motion [15,16]. These limitations severely affect the quality of life of these patients and their participation in daily, social, and professional life. However, the specific biomechanical alterations underlying these gait abnormalities remain subject to ongoing debate in the literature. Consequently, achieving robust functional outcomes in mobility following surgical intervention is critical for long-term quality of life.

Although recent systematic reviews have examined lower limb biomechanics in patients with lower extremity bone tumors relative to healthy controls [17], existing analyses neglected the anatomical distinctions by combining results from different joints, and typically focused solely on basic spatiotemporal gait parameters. The knee joint, however, involves coupled rolling and gliding motion, presenting unique biomechanical challenges distinctly different from the hip’s ball-and-socket structure or the ankle’s hinge structure. Therefore, a targeted analysis focusing exclusively on periarticular knee tumors is warranted to derive clinically precise and specific evidence. This systematic review and meta-analysis were designed to: (1) compare the biomechanical profiles in patients undergoing prosthetic reconstruction for distal femur and proximal tibia tumors with those of contralateral limbs or healthy controls; and (2) identify persistent postoperative biomechanical deficits. The findings are expected to critically assess the biomechanical effectiveness of the procedure, which will greatly benefit the development of postoperative rehabilitation strategies for patients with lower extremity bone tumors.

## 2. Materials and Methods

### 2.1. Protocol

This study adheres to the Preferred Reporting Items for Systematic Reviews and Meta-Analyses (PRISMA) reporting guideline. Our protocol is registered on PROSPERO (CRD420250655463).

### 2.2. Search Strategy

In accordance with the Population, Intervention, Comparison, Outcome and Study design PICOS framework, a complete search was conducted across Scopus, Embase, PubMed, and Cochrane databases from their inception to January 2025. Full search strategies of each database are available in Tables S1–S4 of the Supplementary Material S1.

### 2.3. Study Selection

The initial screening of articles was conducted independently by two authors (Y.D.G. and Y.C.L.). This process involved a systematic assessment of titles and abstracts using EndNote X9 (Clarivate Analytics, Boston, MA, USA). Subsequently, any studies identified as potentially eligible underwent a full-text review to determine their inclusion in the systematic review. Any discrepancies between the two reviewers were resolved through consultation with a third reviewer (S.H.Y.).

Studies were considered eligible under the PICOS framework if they enrolled patients with distal femoral or proximal tibial tumors treated with endoprosthetic reconstruction, with comparators defined as healthy controls or contralateral limbs. Primary outcomes comprised quantitative assessments of lower-extremity biomechanics such as gait kinematics, kinetic profiles, postural stability, proprioceptive function, and muscle strength. Exclusion criteria were: (1) comorbid lower-extremity musculoskeletal pathologies; (2) non-empirical research (case reports, reviews, abstracts); and (3) non-English publications.

### 2.4. Data Extraction

Data extraction was performed independently by two reviewers (Y.D.G. and Y.C.L.) using a pre-designed Excel spreadsheet (Microsoft, Redmond, Washington, DC, USA). Discrepancies were resolved through consensus discussion. If additional information is required, the corresponding author of the study will be contacted. The data extracted encompassed the first author, publication year, study design, participant number, age, post-operative duration, tumor type, tumor location, prosthesis implant type, and biomechanical parameters (e.g., joint angles, gait velocity). Data were collated in mean  ±  standard deviation (SD) or standard error (SE) format.

### 2.5. Risk of Bias Assessment

Risk of bias was assessed using the Newcastle-Ottawa Scale (NOS) [18,19] for observational studies and the Agency for Healthcare Research and Quality (AHRQ) tool for cross-sectional studies [20,21]. This evaluation was performed by two independent reviewers (Y.D.G. and Y.C.L.). Discrepancies in scoring were resolved through consensus discussion; if unresolved, a third reviewer (S.H.Y.) arbitrated the final decision.

### 2.6. Statistical Analysis

Quantitative synthesis was performed if more than three studies reported the same biomechanical outcome. Outcome measures were all continuous variables. The Standardized Mean Difference (SMD) computed from the raw sample means, standard deviations, and sample sizes extracted from each study was used to pool effect sizes across studies in this meta-analysis. Heterogeneity was assessed using Cochran’s Q statistic and quantified with the I^2^ statistic [22]. Sources of heterogeneity were explored through subgroup analysis. Fixed-effects and random-effects models were applied for meta-analysis based on the degree of heterogeneity. The potential for publication bias was evaluated using funnel plots and the Egger test [23]. Sensitivity analyses were conducted to examine the robustness of the pooled estimates by sequentially excluding individual studies. All statistical analyses were conducted using Stata17 (StataCorp, College Station, TX, USA).

### 2.7. Quality of Evidence

The certainty of evidence was assessed for each primary outcome using the Grading of Recommendations Assessment, Development, and Evaluation (GRADE) approach. The initial quality of evidence was set as low due to the inclusion of observational studies. Evidence was then downgraded based on five factors (risk of bias, inconsistency, indirectness, imprecision, and publication bias) or upgraded based on three factors (large magnitude of effect, dose–response gradient, and confounding).

## 3. Results

**Figure 1:**
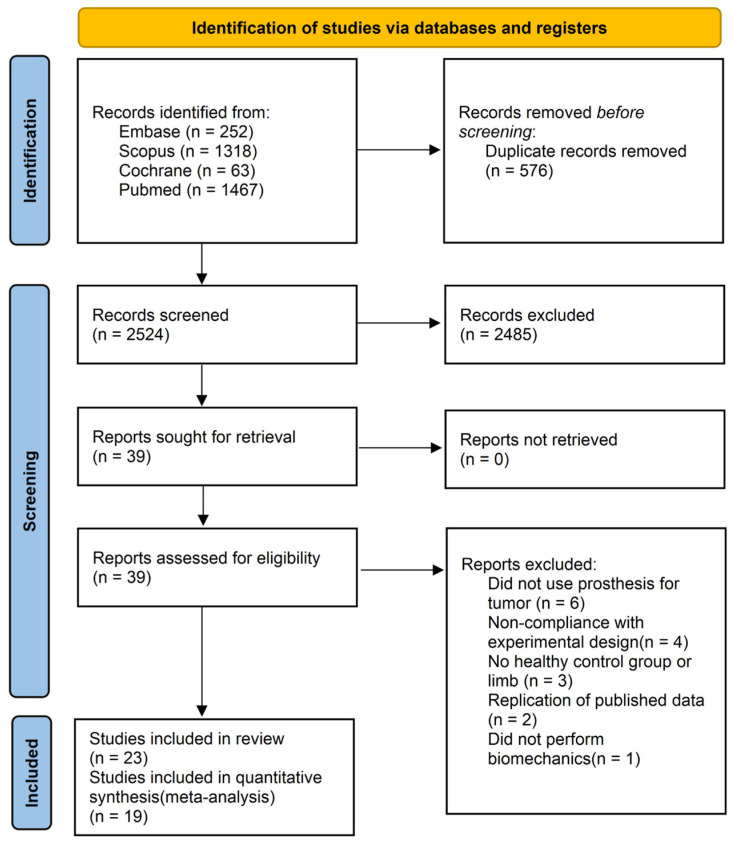
PRISMA 2020 flow diagram for new systematic reviews which included searches of databases and registers only.

Of the 3100 records identified from electronic databases, 3061 records were omitted after removing duplicates and excluding irrelevant studies. Full texts were retrieved for all 39 remaining records and the remaining 23 studies [24,25,26,27,28,29,30,31,32,33,34,35,36,37,38,39,40,41,42,43,44,45,46] met the final inclusion criteria. Due to unavailable data and insufficient literature reporting specific parameters for quantitative analysis, a total of 19 studies were ultimately included in the meta-analysis. The screening process of the study is shown in Figure 1. The characteristics of the included studies are summarized in Table 1.

**Figure 2:**
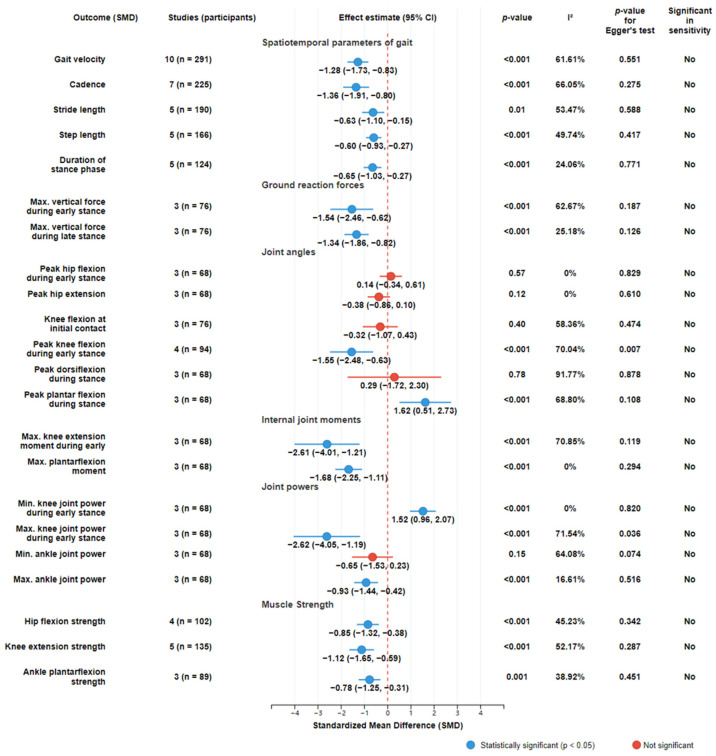
Summary of pooled results. The meta-analysis includes results categorized across six primary domains: Spatiotemporal parameters of gait, Ground reaction forces, Joint angles, Internal joint moments, Joint powers, and Muscle Strength.

The majority of included investigations predominantly utilized retrospective observational designs. Our analysis encompassed a total of 370 patients treated with oncologic knee prostheses and 322 healthy controls. The included studies primarily focused on: gait analysis (n = 15), knee muscle strength (n = 6), energy expenditure during walking (n = 3), physical activity level (n = 3), balance (n = 1), and joint position sense (n = 1). Studies assessing multiple functional domains were systematically classified into corresponding analytical categories. Five studies [31,35,40,42,44] included subgroup comparisons based on tumor location (distal femur versus proximal tibia). The summary of pooled results is shown in Figure 2.

### 3.1. Gait Biomechanics

15 studies evaluated gait biomechanics; however, due to unavailable data [40], quantitative analysis was conducted on 14 articles [16,24,27,28,30,32,33,34,35,36,37,38,40,44]. Outcome measures were categorized into three domains: spatiotemporal parameters, kinematic parameters, and kinetic parameters (Figure 2).

**Figure 3:**
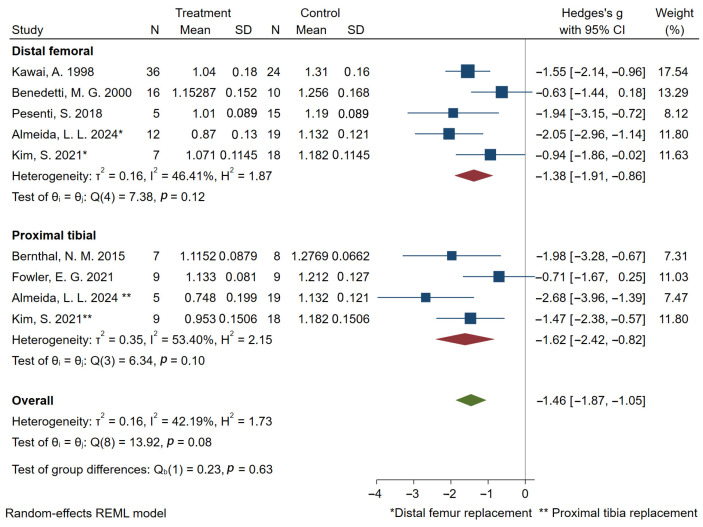
Forest plot of subgroup analysis of gait velocity (m/s) in the distal femur and proximal tibia. Almeida 2024 and Kim 2021 are marked and used twice because distal femoral and proximal tibia were studied separately [40,44].

**Figure 4:**
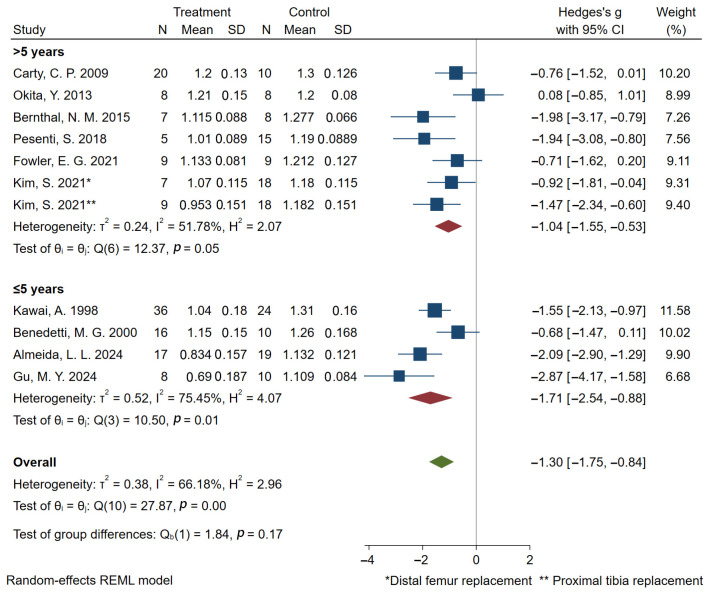
Forest plot of subgroup analysis of gait velocity (m/s) at different postoperative time points. Kim 2021 is marked and used twice because distal femoral and proximal tibia were studied separately [40].

**Figure 5:**
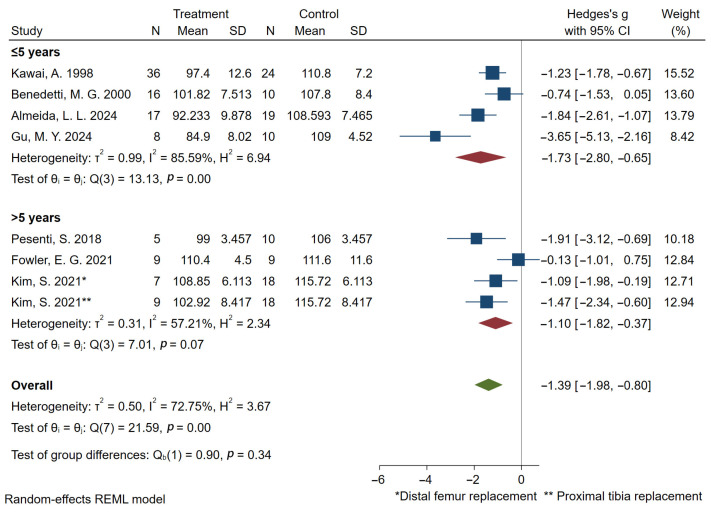
Forest plot of subgroup analysis of cadence (step/min) at different postoperative time points. Kim 2021 is marked and used twice because distal femoral and proximal tibia were studied separately [40].

Regarding gait velocity, the pooled results based on 10 studies [16,27,28,32,33,35,36,38,40,44] showed that prosthetic reconstruction significantly reduced gait velocity statistically. Subgroup analyses based on tumor location indicated significant reductions in gait velocity for both distal femoral and proximal tibial reconstructions (Figure 3). Subjects with a postoperative time of less than five years showed a higher effect size compared to those with a postoperative time of more than five years (Figure 4). 7 studies [16,27,28,36,38,40,44] reported cadence outcomes. Overall, patients exhibited a significant reduction in cadence compared to controls. Subjects with a postoperative time of less than five years exhibited greater deficits in cadence compared to those with a postoperative time of more than five years (Figure 5). And 5 studies reported reduced stride and step length.

5 studies [28,34,37,38,40] reported that the stance phase time of patients with prosthetic reconstruction was significantly reduced. 3 studies [34,37,38] highlighted asymmetry in single-limb support time, with shorter durations on the surgical limb compared to the contralateral side.

3 studies [28,33,40] reported ground reaction force (GRF) outcomes during gait. Overall, significant reductions in peak vertical GRF were observed during both early stance and late stance phases.

4 studies [28,33,38,40] reported joint angles of the hip, knee, and ankle during gait. Meta-analysis results demonstrated statistically significant reductions in peak knee flexion during early stance and increased maximal plantar flexion during stance. No significant differences were observed for other parameters.

3 studies [24,38,40] reported joint internal moments and joint power during gait. Meta-analysis showed significantly reduced maximal knee extension during early stance and plantarflexion moments. For joint power, both peak absorption and generation during early stance demonstrated significant differences. At the ankle, peak power generation was significantly lower in patients, while absorption showed no significant difference.

### 3.2. Muscle Strength

4 studies [30,39,42,43] evaluated isokinetic knee muscle strength following prosthetic reconstruction using angular velocities of 60°/s and 180°/s. Overall, patients with prosthetic reconstruction demonstrated significantly lower isokinetic extension strength and flexion strength compared to controls across both testing velocities (Figure 2).

### 3.3. Energy Cost of Walking and Physical Activity Levels

3 studies [16,27,35] evaluated postoperative walking energy expenditure. Kawai et al. [27] (36 patients, 26-month follow-up) demonstrated that the mean net energy cost (ml O2/m/kg) for patients was 135% of that observed in controls, with the percentage of predicted maximum aerobic capacity reaching 168% (*p* < 0.0001 for both comparisons). Gu et al. [16,44] reported significantly increased energy expenditure (kcal/min/kg) (8 patients, >12-month follow-up). In contrast, Bernthal et al. [35] (15 patients, 162-month follow-up) found no significant differences in oxygen consumption during gait.

Three studies [30,35,38] reported daily step cycle outcomes. A study with 94 months of mean postoperative follow-up [31] revealed significantly reduced daily step cycles compared to healthy controls, whereas another study [38] found no significant differences with 169-month follow-up. Additionally, no statistically significant differences were observed between proximal tibia and distal femur groups at 162.48 months of follow-up [35].

One study [45] reported functional assessments of the Timed Up and Go Test (TUGT) and 6-min walk test (6MWT) in 31 participants at a mean follow-up of 49.3 months. Statistically significant differences were observed for both TUGT (*p* = 0.008) and 6MWT (*p* = 0.002).

**Table 1:** Characteristics of the included studies.

Study	Study Design	Intervention (*n*)	Healthy Controls (*n*)	Age (Mean)	Evaluation Months After Surgery (Mean)	Tumor Type	Location of Tumor	Type of Implant	Outcome Measures
Tsuboyama et al. (1993) [24]	Retrospective	21	4	24	48	Primary malignant bone tumor	Distal femur	Hinged endoprosthesis	Quadriceps muscle mass, Knee extension and flexion strength
Tsuboyama et al. (1994) [25]	Case series	20	0	23	48	Primary malignant bone tumor	Distal femur	Modular uncemented prosthesis	Length of contact phase, peak pressure, and force-time integral of total foot and in various regions under the foot, Knee extension strength
Petschnig et al. (1995) [26]	Retrospective	17	50	29	60	Primary malignant bone tumor	Proximal tibia	Modular cementless prosthesis, Custom-made tumor prosthesis	Knee function scores, Muscle strength, Electromyography (EMG)
Kawai et al. (1998) [27]	Retrospective	36	24	24	26	Osteosarcoma, Ewing sarcoma, Malignant fibrous histiocytoma, Chondrosarcoma, Lymphoma	Distal femur	Lane-Burstein knee prostheses, Finn knee prostheses	Carbon dioxide content, oxygen content, and volume of expired air; Velocity, cadence, stride length, gait-cycle time, and right and left single-limb support times; The maximum voluntary isometric extensor and flexor torque
Benedetti et al. (2000) [28]	Retrospective	16	10	29	44	Osteogenic sarcoma, Malignant fibrohistiocytoma, Fibrosarcoma	Distal femur	Modular and hinged cementless prosthesis (KMFTR)	Maximum extension moment, Foot-ground reaction forces, Kinematic and Kinetic findings, Electromyographic activity
Li et al. (2005) [29]	Case–control	20	20	21.7	36	Osteosarcoma	Distal femur, Proximal tibia	Modular endoprosthesis	Angular repositioning
Tsauo et al. (2006) [30]	Cross-sectional	20	20	21.7	36	Osteosarcoma	Distal femur, Proximal tibia	Modular endoprosthesis	Isokinetic knee muscle strength, Range of motion (ROM) of the knee, Gait and Enneking functional score
Rosenbaum et al. (2008) [31]	Retrospective	22	26	34.5	94	Osteosarcoma, Chondrosarcoma, Ewing’s sarcoma, Malignant fibrous histiocytoma	Distal femur, Proximal tibia	Modular prostheses (mostly MUTARS prosthesis, 20 cases; Kotz prosthesis, 2 cases)	Time spent in different activity categories and movement intensity, Number of step cycles and gait data intensity
Carty et al. (2009) [32]	Retrospective	20	10	16	90	Bone sarcoma	Distal femur, Proximal tibia	Stryker HMRS, Stanmore SMILES	Gait velocity, step width, stance and swing times, joint angles, ground reaction forces, joint moments, joint power
Okita et al. (2013) [33]	Cross-sectional	8	8	30	91	Osteosarcoma, Giant cell tumor, Chondrosarcoma	Distal femur, Proximal tibia	Kyocera Limb Salvage System, Japan Medical Materials K-MAX KNEE System K-5, Howmedica Modular Resection System	Walking speed, Ground reaction forces, Joint angles, Internal joint moments, Joint powers,
AlGheshyan et al. (2015) [34]	Retrospective	5	10	42.25	43.2	Malignant bone tumor	Distal femur	Modular rotating knee system	Cadence, Stride length, Speed, Step length ratio, Stance duration, Joint motion ranges
Bernthal et al. (2015) [35]	Retrospective	15	8	33.7	162.48	Bone sarcoma	Distal femur, Proximal tibia	Howmedica, Techmedica, or Stryker implants with rotating hinge knee components and cemented stems.	Oxygen consumption during gait, Walking speed, Knee extension and flexion strength, Mean number of strides per day
Pesenti et al. (2018) [36]	Retrospective	6	10	24.7	97.2	Osteosarcoma, Ewing’s sarcoma	Distal femur	Constrained megaprosthesis, with fixed hinge and a cemented stem	Gait speed, Stride length, Cadence, Hip flexion extension, Knee flexion extension and ankle flexion extension, Gait Deviation Index (GDI) and the Gillette Gait Index (GGI)
Singh et al. (2018) [37]	Cross-sectional	20	0	22.75	84	Osteosarcoma, Giant cell tumor	Distal femur, Proximal tibia	Rotating hinge knee mechanism and cemented prosthesis	Walking velocity, Stride length, Duration of stance, Goniometry of the knee
Fowler et al. (2021) [38]	Cross-sectional	9	9	31	169.2	Primary malignant bone tumors	Proximal tibia	Kinematic Rotating Hinge knee prosthesis	Gait spatial-temporal data, Joint kinematics Kinetics, Community walking Cadence, knee joint torque
Graulich et al. (2021) [39]	Retrospective	9	0	65	36		Distal femur, Proximal tibia	Modular Universal Tumor and Revision System	Knee extension and flexion force
Kim et al. (2021) [40]	Retrospective	16	18	22.1	63.6	Osteosarcoma, Giant cell tumors, Ewing’s sarcoma	Distal femur, Proximal tibia	Single modular universal tumor and revision system	Ground reaction forces, Joint angles, Reaction joint moments, Joint powers, Spatiotemporal gait parameters (stance phase, swing phase, double support, velocity, stride length, cadence)
Kumar et al. (2021) [41]	Retrospective	7	0	27.6		Osteosarcoma, Ewing’s sarcoma	Proximal tibia		Velocity, Cadence, Swing phase percentage, Single limb support phase percentage, Step lengths, Gait Profile Score
Johansen et al. (2023) [42]	Cross-sectional	18	18	31	91.6	Bone sarcoma	Distal femur, Proximal tibia		Isokinetic muscle strength, Knee joint work capacity, Maximal voluntary contraction and rate of force development
Rodrigues et al. (2023) [43]	Prospective cross-sectional	9	0	45.9	70.8		Distal femur	The Modular Universal Tumor and Revision System	Peak torque in knee extension and flexion
Almeida et al. (2024) [44]	Cross-sectional	17	19	28.9	39	Ewing, Giant cell tumor of bone, Osteosarcoma, Non-oncological lesions with aggressive behavior, Chondroblastoma	Distal femur, Proximal tibia	Fabroni-type endoprosthesis made of polyethylene	Gait velocity, Cadence, Step length, Stance time, Swing time, Moment of force, Power generated, Ground reaction forces
Gu et al. (2024) [16]	Retrospective	8	10	41		Giant cell tumors, Osteosarcoma, Chondrosarcoma	Distal femur, Proximal tibia	Cobalt-chromium-molybdenum or titanium alloy RHK	Walking speed, Cadence, Single support, Swing duration, Energy Expenditure
Jover-Jorge et al. (2024) [45]	Case–control	31	48	39.3	49.3	Osteosarcoma, Chondrosarcoma, Giant cell tumor, Metastasis, Plasmacytoma, Undifferentiated soft tissue sarcoma	Distal femur	Distal femur tumor prosthesis with a conventional intramedullary stem, biological osseointegration system	Timed Up and Go (TUG), 6-min Walk Test (6MWT), Knee flexor and extensor muscle strength

### 3.4. Proprioception and Balance

One study [29] evaluating knee proprioception in 20 patients found no differences in knee proprioception between patients and controls, nor across distal femoral and proximal tibial tumor groups. However, patients with ≥40% resection length had significantly poorer proprioception than those with smaller resections.

One study [34] evaluated the center of pressure (COP) sway in reconstructed limbs at 43.2 months post-reconstruction. Compared to healthy controls, patients exhibited significantly reduced COP sway in the mediolateral direction but greater sway in the anteroposterior direction.

### 3.5. Risk of Bias, Publication Bias, Sensitivity Analyses and Quality of Evidence

Most studies demonstrated moderate to low risk of bias, with few studies rated as moderate risk (Table 2, Table 3 and Table 4).

All funnel plots for assessing publication bias are presented in Supplementary Material S2. Publication bias was detected in Peak knee flexion and Maximum knee joint power during early stance. However, sensitivity analysis employing the trim-and-fill method demonstrated that the adjusted pooled estimates remained consistent with the original results, indicating the robustness of the synthesized effect sizes. Sensitivity analyses, performed by sequentially excluding individual studies, demonstrated consistent effect sizes. The final GRADE ratings and the detailed justifications are presented in the Summary of Findings table located in the Supplementary Material S3.

## 4. Discussion

This study confirmed that endoprosthetic reconstruction after knee tumor resection significantly alters lower-limb biomechanical profiles. Compared to healthy controls or contralateral limbs, endoprosthetic reconstruction following knee tumor resection was associated with marked reductions in all quantified gait parameters and knee muscle strength. Concurrently, a consistent trend toward elevated energy expenditure in walking and reduced physical activity levels was observed.

### 4.1. Gait

Compared to the contralateral limb or healthy controls, patients undergoing tumor resection with prosthetic reconstruction exhibited marked reductions in key gait parameters, including gait velocity, cadence, stride length, step length, and stance phase duration.

Notably, subgroup analyses stratified by postoperative follow-up duration revealed that patients with follow-up periods ≤5 years exhibited greater deficits in gait velocity and cadence compared to controls. This disparity suggested that patients in the early adaptive phase faced significant mechanical challenges. Specifically, their slower, less rhythmic gait was a compensation for reduced knee extensor and flexor strength. As these patients lacked full active knee control, reducing speed was a necessary strategy to enhance stability and ensure safe limb swing. In contrast, the deficit was mitigated in long-term follow-up cohorts (>5 years), likely due to the development of robust neuromuscular compensatory mechanisms alongside the functional benefits of sustained rehabilitation. These long-term gains reflected enhanced muscle strength and a resultant increase in knee joint stability, allowing patients to achieve gait parameters closer to those of the control group.

Furthermore, tumor location may influence functional outcomes, with proximal tibial reconstructions associated with marginally reduced gait velocity than distal femoral reconstructions. This phenomenon may originate from the necessity for concomitant extensor mechanism reconstruction during proximal tibial tumor resection and prosthetic implantation. Interestingly, only Kim et al. and Almeida et al. conducted comparative kinematic analyses stratifying by tumor location, yet revealed conflicting conclusions. Kim et al. stated that distal femur group demonstrated greater hip extension and knee extension in the stance phase, whereas proximal tibia group was the one that demonstrated greater hip flexion and knee flexion. Contrastingly, Almeida et al. documented hip flexion patterns in both groups, with proximal tibial cases demonstrating sustained knee hyperextension during stance. These discrepancies may reflect limitations in statistical power due to restricted cohort sizes across studies.

GRF analysis demonstrated reduced peak vertical GRF during both early and late stance phases, suggesting a stiff-knee gait strategy characterized by diminished knee flexion and impaired muscle function. This interpretation was biomechanically corroborated by diminished peak knee flexion angles and reduced internal extension moments during early stance. Increased peak ankle plantar flexion angles during the early stance phase may reflect compensatory adaptation to quadriceps insufficiency. In cases of quadriceps weakness, this deficiency was compensated for by the hip extensor muscles, which subsequently aided in placing the lower limb in a more extended position. This positioning shifted the body’s center of gravity anterior to the knee joint, thereby reducing the flexion moment and effectively “locking” the knee joint to maintain stability. Furthermore, meta-analysis results demonstrated reductions in peak internal plantar flexion moments and ankle power generation, suggesting composite deficits in the calf muscles in addition to quadriceps insufficiency.

### 4.2. Muscle Strength

All four included studies reported reduced knee extensor and flexor strength across both high- and low-velocity isokinetic testing protocols. Notably, studies highlighted significantly greater deficits in knee extension and flexor strength among patients with proximal tibial tumors compared to those with distal femoral tumors [35,46]. Previous systematic reviews on Fitness, Function, and Exercise Training Responses have also identified impairments of lower limb strength and flexibility following limb salvage with a lower limb megaprosthesis [47]. These findings may be attributed to the weakened knee extensor mechanism.

### 4.3. Energy Cost of Walking and Physical Activity Levels

In the domain of energy expenditure and physical activity levels, although some studies reported no statistically significant differences, all observed trends toward elevated energy consumption and reduced daily step counts in patients compared to healthy controls. These non-significant trends likely reflect heterogeneity in postoperative follow-up intervals, with long-term cohorts exhibiting relatively normalized gait energy demands and activity levels [33]. Elevated energy expenditure and reduced physical activity levels during the early postoperative period may be attributed to postoperative catabolic states, pain and impaired muscle function. Following prosthetic reconstruction, an altered gait pattern is frequently adopted to mitigate pain and compensate for joint instability, resulting in diminished energy efficiency of walking [48]. With prolonged recovery intervals, progressive neuromuscular adaptations and compensatory mechanisms facilitate the restoration of normative gait energetics. Notably, several studies [49,50] in the literature indicate that targeted exercise interventions can yield positive effects on lower limb gait function in patients with endoprosthetic reconstruction for distal femur and proximal tibia tumors. Therefore, implementing structured exercise programs is clinically necessary to effectively enhance patient mobility, improve walking efficiency, and ultimately increase their physical activity levels and long-term quality of life.

### 4.4. Proprioception and Balance

Postoperative knee joint position sense in the operated limb showed no significant differences compared to the contralateral side [29]. Interestingly, proprioception appeared unaffected by tumor location, despite substantial differences in resected muscle volume between groups. This finding has two possible explanations: First, the bone tumor incidence population is generally younger, making it easier to develop proprioceptive compensatory mechanisms post-surgery, which restores the affected side’s proprioception to the level of the healthy side. Second, the proprioception on the healthy side may have deteriorated, as was found by Nemati et al. [51] in their study. Roberts et al. [52] hypothesized that aberrant afferent information from receptors in one limb would affect the receptors in the contralateral limb. In contrast, resection length emerged as a critical determinant. The proprioception of the patients with a greater length of bone resected was significantly worse in both lower extremities than that of patients with a shorter resected length. Regarding postural stability, patients demonstrated increased anteroposterior COP sway during quiet standing, suggesting impaired sagittal plane dynamic control. This may be attributed to quadriceps weakness or prosthetic design constraints on sagittal plane mobility.

### 4.5. Clinical Implications and Recommendations

Our study elucidated persistent deficits in gait patterns, muscle strength, mobility, and proprioception in the biomechanical outcomes of endoprosthetic reconstruction for distal femur and proximal tibia bone tumors through comparative analysis of patient cohorts and healthy controls. These findings offered clinicians an evidence-based framework to address postoperative functional impairments and optimize rehabilitation strategies. Our findings emphasized the clinical imperative to prioritize rehabilitation interventions targeting abnormal gait mechanics, reduced knee flexion and extension strength, and sagittal plane instability. These impairments are consistently linked to biomechanical disruptions that limit motor function, as demonstrated in multiple studies.

### 4.6. Strengths and Limitations

To our knowledge, this is the first systematic review and meta-analysis to rigorously evaluate the biomechanical consequences of endoprosthetic reconstruction in patients with distal femoral and proximal tibial bone tumors. Therefore, a targeted analysis focusing exclusively on distal femur and proximal tibia tumors is warranted to derive clinically precise and specific evidence. Notably, in contrast to Filis et al. (2022) [17], which focused primarily on basic gait parameters, this study provided a comprehensive assessment of lower limb biomechanical outcomes across six key domains: gait analysis parameters, knee muscle strength, energy expenditure during walking, physical activity level, balance, and joint position sense. This comprehensive scope establishes the novelty and unique value of our review.

However, the rarity of knee bone tumors introduces limitations to this review. The paucity of eligible studies for some of the meta-analyzed outcomes necessitated cautious interpretation of the pooled estimates. Restricted cohort sizes precluded robust quantitative synthesis of balance, energy expenditure, and other parameters, mandating future large-scale investigations for validation. Additionally, due to resource and translation constraints, this review was restricted to English-language publications. We acknowledge that this exclusion may introduce language bias, and recommend that future reviews perform multi-lingual searches for more comprehensive evidence.

### 4.7. Future Research Directions

Our systematic review highlighted the pressing need for future high-quality, prospective studies with standardized protocols. Future research should prioritize subgroup analyses stratifying distal femoral and proximal tibial cohorts, as this approach may yield clinically significant insights given their divergent intraoperative muscle resection patterns and distinct biomechanical environments.

Furthermore, the impact of various endoprosthetic designs on long-term function remains unclear. Different implant types and materials likely confer distinct effects on the mechanics and stability of the lower limb. Therefore, a critical direction for future investigation is to specifically evaluate and compare the long-term biomechanics of the lower limb across different prosthetic designs to determine which implant type offers superior functional outcomes and durability.

## 5. Conclusions

Characterizing persistent postoperative biomechanical and physical performance deficits becomes increasingly critical since survival rates and survival duration for distal femur and proximal tibia tumors are predicted to rise with advances in oncology, thereby necessitating targeted rehabilitation protocols to optimize functional recovery. Overall, our study demonstrated that endoprosthetic reconstruction after distal femur and proximal tibia bone tumor resection significantly altered the biomechanical parameters of patients. However, these findings should not be misconstrued as opposing the established benefits of prosthetic reconstruction for knee tumor patients, but instead underscore substantial potential for improvement in both surgical techniques and postoperative rehabilitation strategies to address persistent functional deficits.

## Figures and Tables

**Table 2:** Quality of cohort studies using the Newcastle-Ottawa Scale (NOS).

Study	Selection				Comparability	Outcome		
	S1	S2	S3	S4		O1	O2	O3
Tsuboyama et al. (1993) [24]	*		*	*	**	*	*	
Petschnig et al. (1995) [26]	*		*	*	**	*	*	
Kawai et al. (1998) [27]	*		*	*	*	*	*	*
Benedetti et al. (2000) [28]	*		*	*	*	*	*	*
Rosenbaum et al. (2008) [31]	*		*	*		*	*	*
Carty et al. (2009) [32]	*		*	*		*	*	*
AlGheshyan et al. (2015) [34]	*		*	*		*	*	*
Bernthal et al. (2015) [35]	*	*	*	*		*	*	
Pesenti et al. (2018) [36]	*		*	*		*	*	*
Graulich et al. (2021) [39]	*	*	*	*	**	*	*	
Kim et al. (2021) [40]	*	*	*	*		*	*	
Kumar et al. (2021) [41]	*	*	*	*	*	*	*	*
Gu et al. (2024) [16]	*	*	*	*		*	*	*

Selection: S1 = Representativeness of the exposed cohort; S2 = Selection of the non-exposed cohort; S3 = Ascertainment of exposure; S4 = Demonstration that outcome of interest was not present at start of study. Outcome: O1 = Assessment of outcome; O2 = Was followed up long enough for outcomes to occur; O3 = Adequacy of follow up of cohorts; * = Represents one point awarded for satisfying a quality criterion; ** = Represents two points awarded for satisfying a quality criterion.

**Table 3:** Quality of case–control studies using the Newcastle-Ottawa Scale (NOS).

Study	Selection				Comparability	Exposure		
	S1	S2	S3	S4		E1	E2	E3
Tsuboyama et al. (1994) [25]	*		*	*	**	*	*	
Li et al. (2005) [29]	*	*	*	*	*	*	*	*
Jover-Jorge et al. (2024) [45]	*	*	*	*	*	*	*	

Selection: S1 = Is the case definition adequate; S2 = Representativeness of the cases; S3 = Selection of Controls; S4 = Definition of Controls. Exposure: E1 = Ascertainment of exposure; E2 = Same method of ascertainment for cases and controls; E3 = Non-Response Rate; * = Represents one point awarded for satisfying a quality criterion; ** = Represents two points awarded for satisfying a quality criterion.

**Table 4:** Quality of cross-sectional studies using the Agency for Healthcare Research and Quality (AHRQ) Recommended Criteria.

Study	1	2	3	4	5	6	7	8	9	10	11
Tsauo et al. (2006) [30]	Yes	Yes	Yes	Yes	Unclear	Yes	Yes	Yes	No	Yes	Not Applicable
Okita et al. (2013) [33]	Yes	Yes	No	No	Unclear	Yes	Yes	Yes	No	Yes	Not Applicable
Singh et al. (2018) [37]	Yes	Yes	No	No	Yes	Yes	Yes	Yes	No	Yes	Not Applicable
Fowler et al. (2021) [38]	Yes	Yes	No	No	Unclear	Yes	No	Yes	No	Yes	Not Applicable
Johansen et al. (2023) [42]	Yes	Yes	Yes	Yes	Unclear	Yes	Yes	Yes	No	Yes	Not Applicable
Rodrigues et al. (2023) [43]	Yes	Yes	Yes	Yes	Unclear	Yes	Yes	Yes	No	Yes	Not Applicable
Almeida et al. (2024) [44]	Yes	Yes	Yes	Yes	Unclear	Yes	Yes	Yes	No	Yes	Not Applicable

1 = Define the source of information (survey, record review); 2 = List inclusion and exclusion criteria for exposed and unexposed subjects (cases and controls) or refer to previous publications; 3 = Indicate time period used for identifying patients; 4 = Indicate whether or not subjects were consecutive if not population-based; 5 = Indicate if evaluators of subjective components of study were masked to other aspects of the status of the participants; 6 = Describe any assessments undertaken for quality assurance purposes; 7 = Explain any patient exclusions from analysis; 8 = Describe how confounding was assessed and/or controlled; 9 = If applicable, explain how missing data were handled in the analysis; 10 = Summarize patient response rates and completeness of data collection; 11 = Clarify what follow-up, if any, was expected and the percentage of patients for which incomplete data or follow-up was obtained.

## Data Availability

The original contributions presented in the study are included in the article material, further inquiries can be directed to the corresponding author.

## Supplementary Materials

The following supporting information can be downloaded at: https://www.mdpi.com/article/10.3390/bioengineering12121310/s1, Supplementary Material S1: Table S1: Search strategy of PubMed; Table S2: Search strategy of Cochrane; Table S3: Search strategy of Embase; Table S4: Search strategy of Scopus. Supplementary Material S2: Figure S1. Funnel plot of gait velocity; Figure S2. Funnel plot of cadence; Figure S3. Funnel plot of stride length; Figure S4. Funnel plot of step length; Figure S5. Funnel plot of duration of stance phase; Figure S6. Funnel plot of maximal vertical force during early stance; Figure S7. Funnel plot of maximal vertical force during late stance; Figure S8. Funnel plot of Peak hip flexion during early stance; Figure S9. Funnel plot of Peak hip extension; Figure S10. Funnel plot of knee flexion at initial contact; Figure S11. Funnel plot of maximal knee flexion during early stance; Figure S12. Funnel plot of peak dorsiflexion during stance; Figure S13. Funnel plot of peak plantar flexion during stance; Figure S14. Funnel plot of max. knee extension moment during early; Figure S15. Funnel plot of max. plantarflexion moment; Figure S16. Funnel plot of min. knee joint power during early stance; Figure S17. Funnel plot of max. knee joint power during early stance; Figure S18. Funnel plot of min. ankle joint power; Figure S19. Funnel plot of max. ankle joint power; Figure S20. 60°/s isokinetic knee extension strength; Figure S21. 60°/s isokinetic knee flexion strength; Figure S22. 180°/s isokinetic knee extension strength; Figure S23. 180°/s isokinetic knee flexion strength. Supplementary Material S3: GRADE evidence profile.

## Abbreviations

The following abbreviations are used in this manuscript:

GRF	Ground reaction force
TUGT	Timed Up and Go Test
6MWT	6-min walk test
COP	Center of pressure
