# Supplementary material for: Lower Limb Biomechanical Outcomes Following Endoprosthetic Reconstruction for Distal Femur and Proximal Tibia Bone Tumors: A Systematic Review and Meta-Analysis
Source: Bioengineering (Basel). 2025 Nov 28;12(12):1310. doi: 10.3390/bioengineering12121310 (PMC12730080; doi:10.3390/bioengineering12121310)
Supplement: Supplementary file 1 [file bioengineering-12-01310-s001.zip › Supplementary Material S1.pdf]

Supplementary Table S1 Search strategy of PubMed

Search number	Query	Results
#1	"Arthroplasty, Replacement, Knee"[Mesh]	34,802
#2	(Arthroplasty, KneeReplacement) OR (Arthroplasties, Replacement, Knee) OR (kneereplacement arthroplasty) OR (knee replacement arthroplasties) OR (Replacement Arthroplasties, Knee) OR (Replacement, Total Knee) OR(Knee Replacement) OR (Knee Arthroplasty) OR (Knee Prosthesis)	64,901
#3	"Prostheses and Implants"[Mesh]	614,884
#4	Implants and Prostheses or Prostheses and Implant or Implant and Prostheses or Prosthetic Implant or Implant, Prosthetic or Implants, Prosthetic or Prosthetic Implants or Implants, Artificial or Artificial Implant or Artificial Implants or Implant, Artificial or Endoprosthesis or Endoprostheses or Prostheses or Prosthesis	696,659
#5	#1 OR #2 OR #3 OR #4	734,962
#6	"Bone Neoplasms"[Mesh]	140,210
#7	(Bone Neoplasm) OR (Neoplasm, Bone) OR (Neoplasms, Bone) OR (Bone Cancer) OR (Cancer of Bone) OR (Cancer of the Bone) OR (chondrosarcoma) OR (Neoplasms) OR(Neoplasms)	4,195,503
#8	#6 OR #7	4,195,503
#9	("Proprioception"[Mesh]) OR "Biomechanical Phenomena"[Mesh]	193,281
#10	(Proprioception) OR (Position Sense) OR (Sense, Position) oR(Posture Sense) OR (Sense, Posture) OR (Sense of Position) OR(Vestibular Sense) OR (Equilibrium Sense) OR (Sense of Equilibrium)OR (biomechanical phenomena) OR (Biomechanics) oR(Biomechanic) oR (Kinematics) oR (kinetic*) oR (MechanobiologicalPhenomena) OR (Phenomena, Biomechanical) OR (gait) OR (torque)OR (angle) OR (strength) OR (force) OR (velocity) OR (moment)	1,757,890
#11	#9 OR #10	1,778,165
#12	#5 AND #8 AND #11	1,467

Supplementary Table S2 Search strategy of Cochrane

Search number	Query	Results
#1	MeSH descriptor: [Arthroplasty, Replacement, Knee] explode all trees	4,125
#2	(Arthroplasty, Replacement, Knee or Arthroplasties, Replacement, Knee or Arthroplasty, Knee Replacement or Prosthetic reconstruction or prosth* or prosthesis or megaprosthesis or endoprosthesis or Knee Replacement Arthroplasties or Knee Replacement Arthroplasty or Replacement Arthroplasties, Knee or Replacement Arthroplasty, Knee or Replacement, Total Knee or Total Knee Replacement or Knee Replacement, Total or Knee Arthroplasty or Arthroplasty, Knee or Arthroplasties, Knee Replacement or Knee Arthroplasty, Total or Arthroplasty, Total Knee or Total Knee Arthroplasty):ti,ab,kw (Word variations have been searched)	29,627
#3	#1 or #2	29,627
#4	MeSH descriptor: [Bone Neoplasms] explode all trees	1,837
#5	(Bone Neoplasms or Bone Neoplasm or Neoplasm, Bone or Neoplasms, Bone or Bone Cancer or Cancer of Bone or Cancer of the Bone or cancer or Neoplasm):ti,ab,kw	227,767
#6	#4 or #5	227,877
#7	MeSH descriptor: [Proprioception] explode all trees	5,279
#8	(proprioception or Position Sense or Sense, Position or Posture Sense or Sense, Posture or Sense of Position or Sense of Equilibrium or Labyrinthine Sense or Vestibular Sense or Sense, Vestibular or Sense, Labyrinthine or Equilibrium Sense):ti,ab,kw	3,501
#9	MeSH descriptor: [Biomechanical Phenomena] explode all trees	4,397
#10	(Biomechanical Phenomena or Phenomena, Biomechanical or Biomechanic Phenomena or Biomechanic Phenomenas or Phenomena, Biomechanic or Phenomenas, Biomechanic or Biomechanics or Biomechanic or Kinematics or Mechanobiological Phenomena or Phenomena, Mechanobiological or biomechanic* or kinematic* or kinetic* or Torque or Force or Gait or Moment or Joint load or Strength or Force or Velocity):ti,ab,kw	121,173
#11	#7 or #8 or #9 or #10	124,707
#12	#3 and #6 and #11	63

Supplementary Table S3 Search strategy of Embase

Search number	Query	Results
#1	'knee replacement'/exp OR 'knee replacement'	52,461
#2	'arthroplasty, replacement, knee':ti,ab,kw OR 'knee joint replacement':ti,ab,kw OR 'knee joint replacements':ti,ab,kw OR 'knee replacement arthroplasty':ti,ab,kw OR 'knee replacements':ti,ab,kw OR 'knee replacement':ti,ab,kw	18,364
#3	#1 OR #2	53,391
#4	('artificial implant':ti,ab,kw OR 'artificial implants':ti,ab,kw OR 'attached prosthetic device':ti,ab,kw OR biodesign:ti,ab,kw) AND endoprosthesis:ti,ab,kw OR 'endoprothesis':ti,ab,kw OR 'implant, artificial':ti,ab,kw OR 'implanted prosthetic device':ti,ab,kw OR 'implants, artificial':ti,ab,kw OR 'internal prosthesis':ti,ab,kw OR 'prosthesis, internal':ti,ab,kw OR 'prosthetic implant':ti,ab,kw OR 'prosthetic implant -retired-':ti,ab,kw OR 'prosthetic implants':ti,ab,kw OR 'endoprosthesis':ti,ab,kw	8,684
#5	'endoprosthesis'/exp	390,050
#6	#3 OR #4 OR #5	432,038
#7	'bone tumor'/exp	242,751
#8	'bone neoplasia':ti,ab,kw OR 'bone neoplasm':ti,ab,kw OR 'bone neoplasms':ti,ab,kw OR 'bone tumorigenesis':ti,ab,kw OR 'bone tumour':ti,ab,kw OR 'neoplasm of the bone':ti,ab,kw OR 'neoplasms of the bone':ti,ab,kw OR 'neoplasms of the bones':ti,ab,kw OR 'neoplastic bone':ti,ab,kw OR 'osseous neoplasm':ti,ab,kw OR 'osseous tumor':ti,ab,kw OR 'osseous tumour':ti,ab,kw OR 'skeletal tumor':ti,ab,kw OR 'skeletal tumour':ti,ab,kw OR 'skeleton tumor':ti,ab,kw OR 'skeleton tumour':ti,ab,kw OR 'tumor of the bone':ti,ab,kw OR 'tumor of the skeleton':ti,ab,kw OR 'tumor, bone':ti,ab,kw OR 'tumor, skeleton':ti,ab,kw OR 'tumour of the bone':ti,ab,kw OR 'tumour, bone':ti,ab,kw OR 'tumour, skeleton':ti,ab,kw OR 'bone tumor':ti,ab,kw	14,117
#9	'chondrosarcoma'/exp	15,049
#10	'cartilage sarcoma':ti,ab,kw OR 'chondroblastic sarcoma':ti,ab,kw OR 'chondromucosarcoma':ti,ab,kw OR 'fibrochondrosarcoma':ti,ab,kw OR 'sarcoma, chondroblastic':ti,ab,kw OR 'chondrosarcoma':ti,ab,kw	11,052
#11	#7 OR #8 OR #9 OR #10	247,270
#12	'proprioception'/exp	17,362

Supplementary Table S3 (continued)

Search number	Query	Results
#13	'deep sensitivity':ti,ab,kw OR 'discrimination, kinaesthetic':ti,ab,kw OR 'discrimination, kinesthetic':ti,ab,kw OR 'kinaesthetic discrimination':ti,ab,kw OR 'kinaesthetic perception':ti,ab,kw OR 'kinesio perceptual test':ti,ab,kw OR 'kinesthetic discrimination':ti,ab,kw OR 'kinesthetic perception':ti,ab,kw OR 'kinetic tonic pattern':ti,ab,kw OR 'muscle propriocepsis':ti,ab,kw OR 'perception, kinaesthetic':ti,ab,kw OR 'perception, kinesthetic':ti,ab,kw OR 'propioception':ti,ab,kw OR 'propriocepsis':ti,ab,kw OR 'proprioceptive innervation':ti,ab,kw OR 'proprioception':ti,ab,kw	10,493
#14	'biomechanics'/exp	144,354
#15	'biomechanical phenomena':ti,ab,kw OR 'biomechanical phenomenon':ti,ab,kw OR 'biomechanism':ti,ab,kw OR 'lifting':ti,ab,kw OR 'biomechanics':ti,ab,kw	68,605
#16	'kinematics':ti,ab,kw OR 'gait':ti,ab,kw OR 'torque':ti,ab,kw OR 'velocity':ti,ab,kw OR 'strength':ti,ab,kw	868,208
#17	#12 OR #13 OR #14 OR #15 OR #16	1,007,103
#18	#6 AND #11 AND #17	252

Supplementary Table S4 Search strategy of Scopous

Search number	Query	Results
#1	TITLE-ABS-KEY (proprioception OR "position sense" OR "sense, position" OR "posture sense" OR "sense, posture" OR "sense of position" OR "sense of equilibrium" OR "labyrinthine sense" OR "vestibular sense" OR "sense, vestibular" OR "sense, labyrinthine" OR "equilibrium sense" OR "biomechanical phenomena" OR "phenomena, biomechanical" OR "biomechanic phenomena" OR "biomechanic phenomenas" OR "phenomena, biomechanic" OR "phenomenas, biomechanic" OR "biomechanics or biomechanic" OR kinematics OR "mechanobiological phenomena" OR "phenomena, mechanobiological" OR biomechanic* OR kinematic* OR kinetic* OR torque OR force OR gait OR moment OR "joint load" OR strength OR force OR velocity)	8,563,915
#2	TITLE-ABS-KEY ("arthroplasty, replacement, knee" OR "arthroplasties, replacement, knee" OR "arthroplasty, knee replacement" OR "prosthetic reconstruction" OR prosth* OR prosthesis OR megaprosthesis OR endoprosthesis OR "knee replacement arthroplasties" OR "knee replacement arthroplasty" OR "replacement arthroplasties, knee" OR "replacement arthroplasty, knee" OR "replacement, total knee" OR "total knee replacement" OR "knee replacement, total" OR "knee arthroplasty" OR "arthroplasty, knee" OR "arthroplasties, knee replacement" OR "knee arthroplasty, total" OR "arthroplasty, total knee" OR "total knee arthroplasty")	555,941
#3	TITLE-ABS-KEY ("Bone Neoplasms" OR "Bone Neoplasm" OR "Neoplasm, Bone" OR "Neoplasms, Bone" OR "Bone Cancer" OR "Cancer of Bone" OR "Cancer of the Bone" OR cancer OR neoplasm OR chondrosarcoma)	5,259,929
#4	#1 AND #2 AND #3	1,318
