# Supplementary material for: Lower Limb Biomechanical Outcomes Following Endoprosthetic Reconstruction for Distal Femur and Proximal Tibia Bone Tumors: A Systematic Review and Meta-Analysis
Source: Bioengineering (Basel). 2025 Nov 28;12(12):1310. doi: 10.3390/bioengineering12121310 (PMC12730080; doi:10.3390/bioengineering12121310)
Supplement: Supplementary file 1 [file bioengineering-12-01310-s001.zip › Supplementary Material S2.pdf]

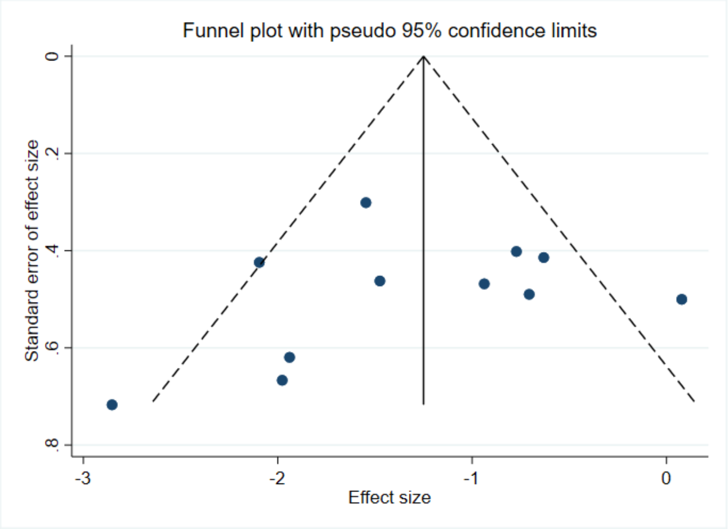

Figure S1. Funnel plot of gait velocity

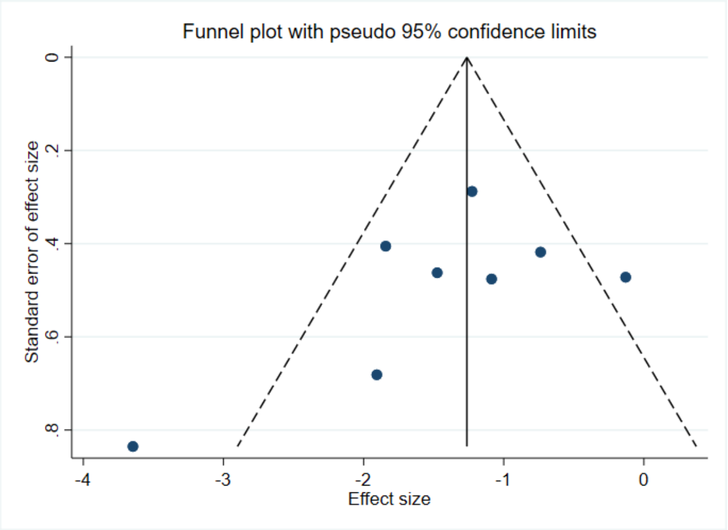

Figure S2. Funnel plot of cadence

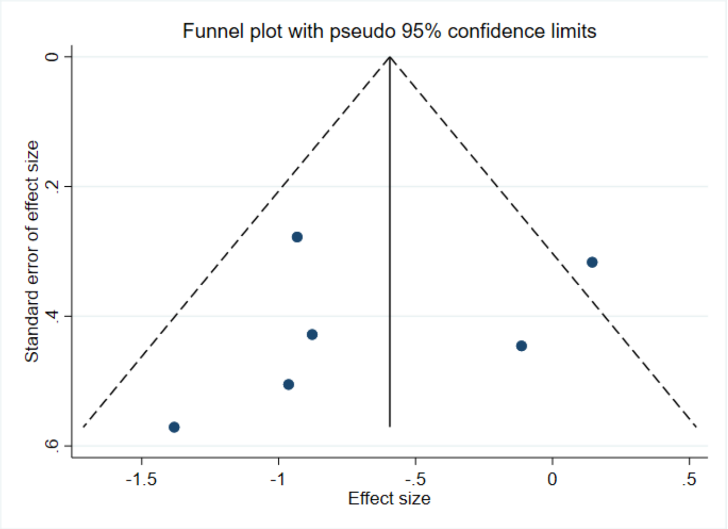

Figure S3. Funnel plot of stride length

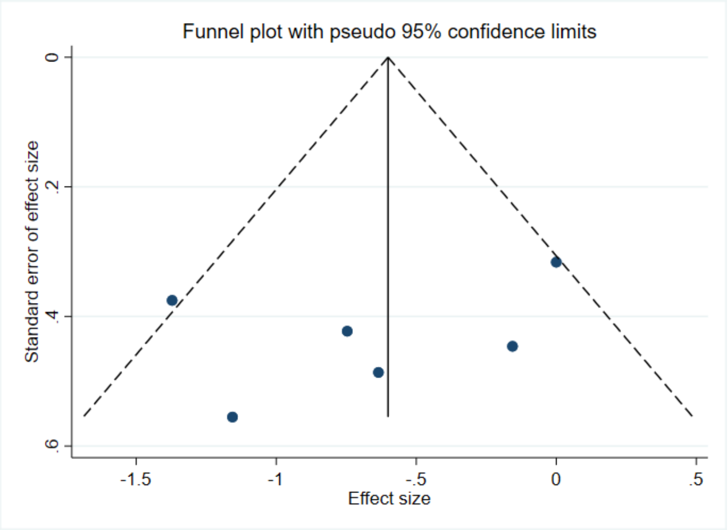

Figure S4. Funnel plot of step length

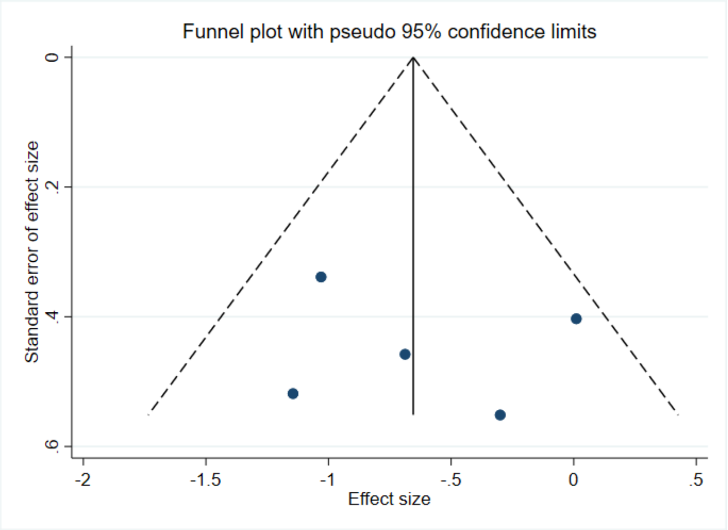

Figure S5. Funnel plot of duration of stance phase

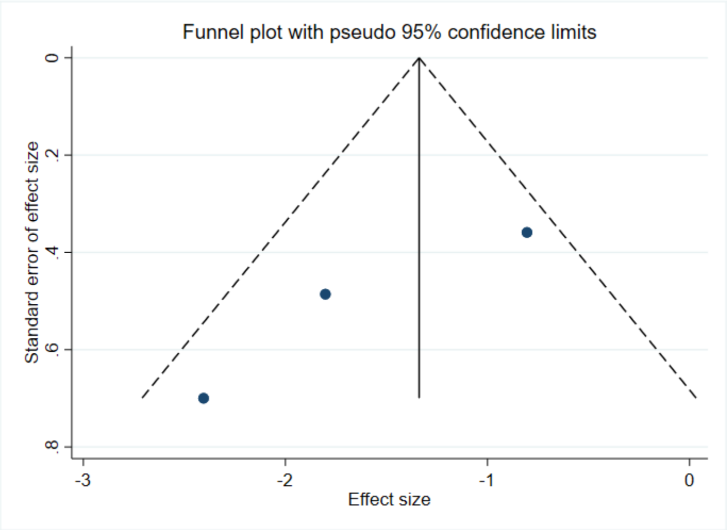

Figure S6. Funnel plot of maximal vertical force during early stance

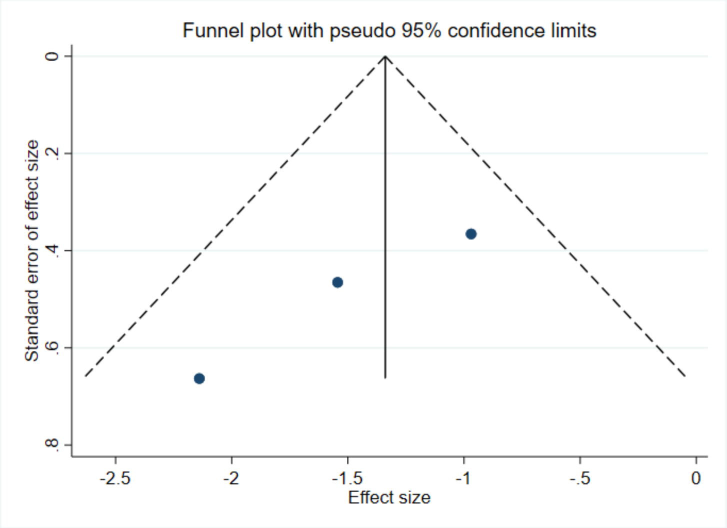

Figure S7. Funnel plot of maximal vertical force during late stance

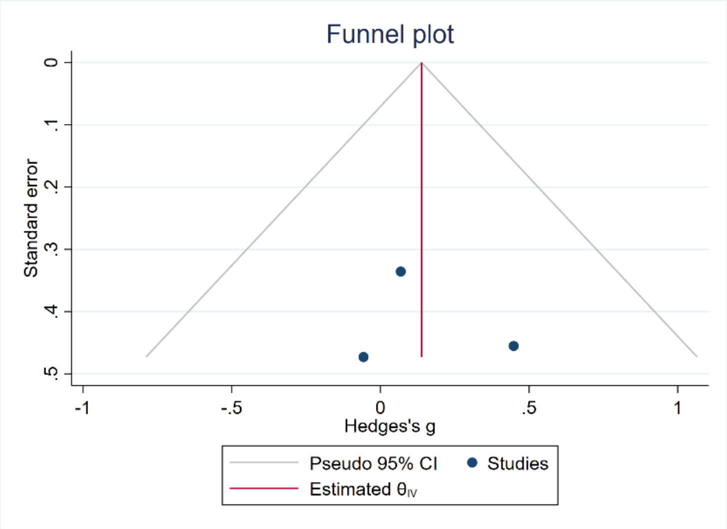

Figure S8. Funnel plot of Peak hip flexion during early stance

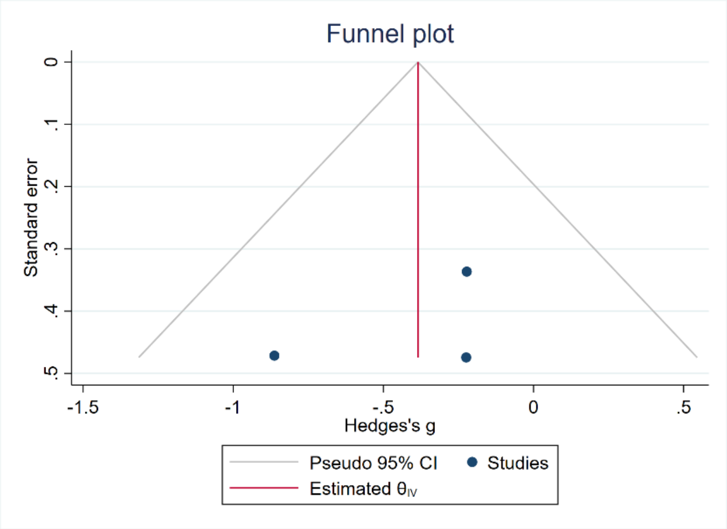

Figure S9. Funnel plot of Peak hip extension

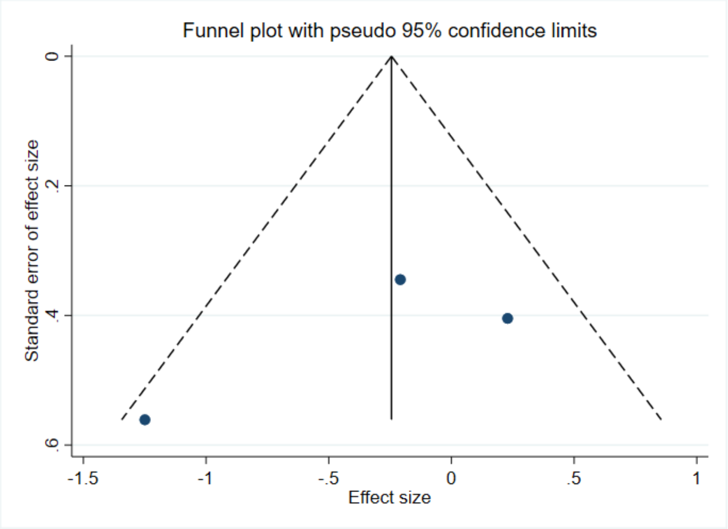

Figure S10. Funnel plot of knee flexion at initial contact

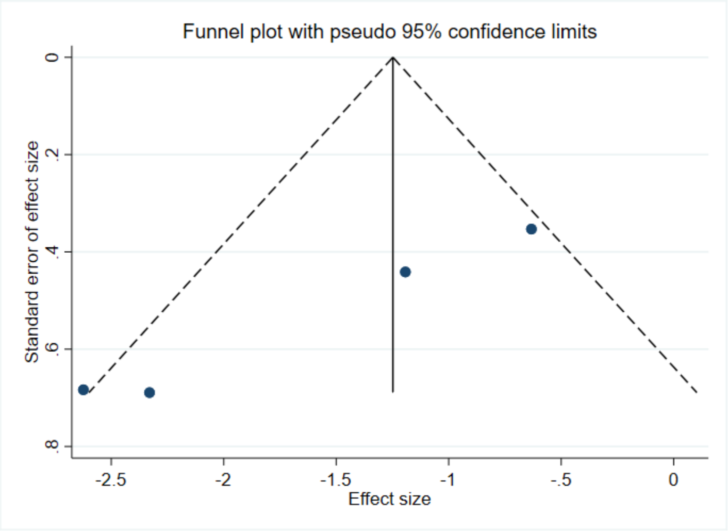

Figure S11. Funnel plot of maximal knee flexion during early stance

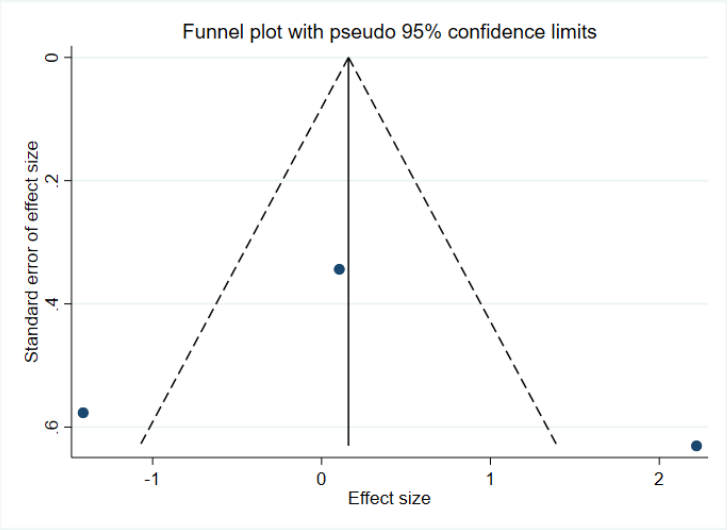

Figure S12. Funnel plot of peak dorsiflexion during stance

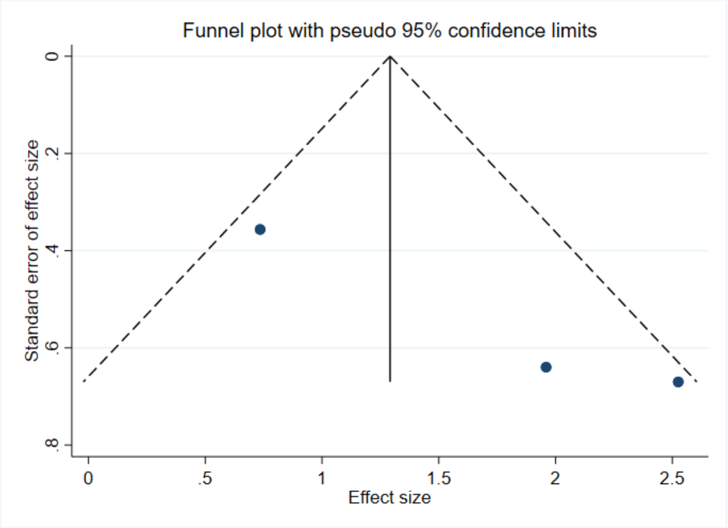

Figure S13. Funnel plot of peak plantar flexion during stance

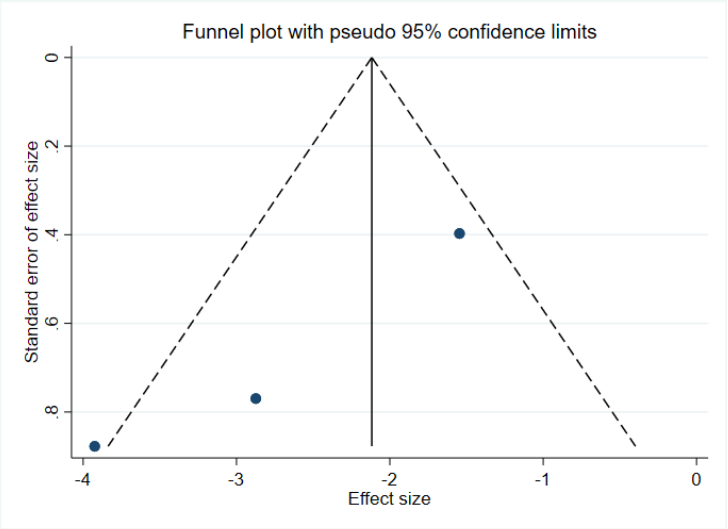

Figure S14. Funnel plot of max. knee extension moment during early

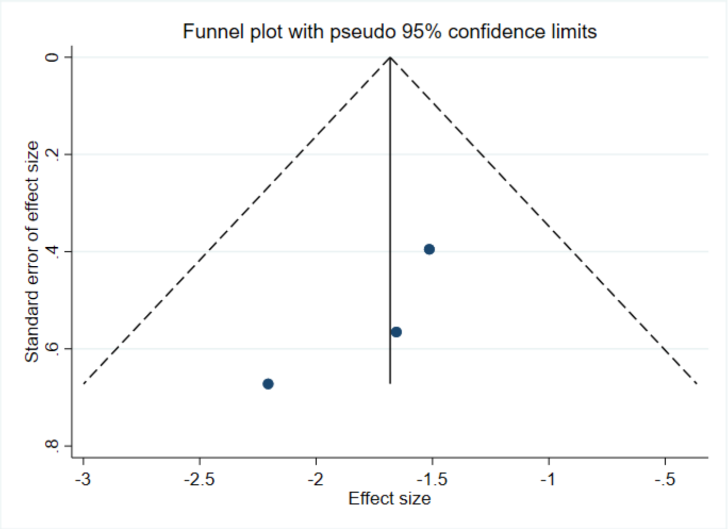

Figure S15. Funnel plot of max.plantarflexion moment

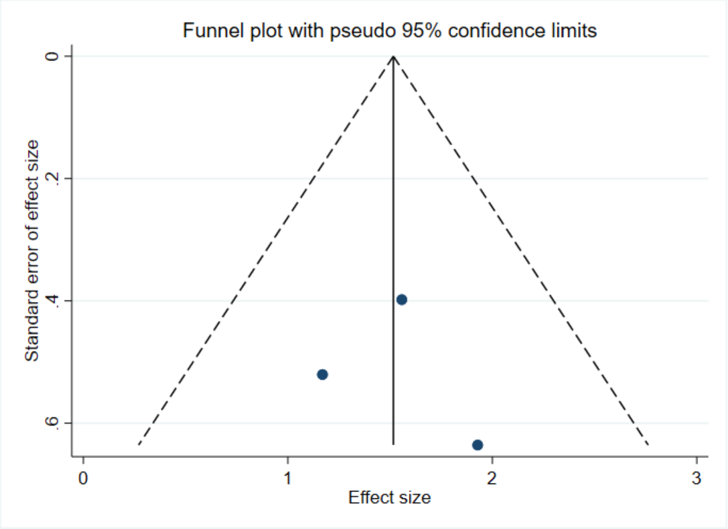

Figure S16. Funnel plot of min. knee joint power during early stance

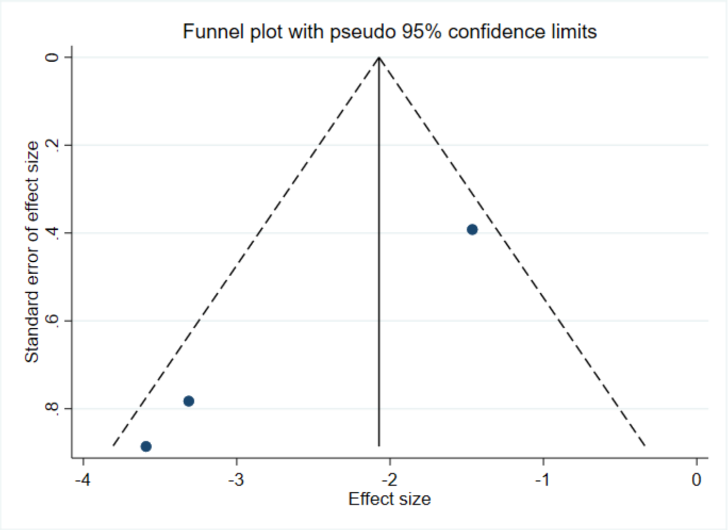

Figure S17. Funnel plot of max. knee joint power during early stance

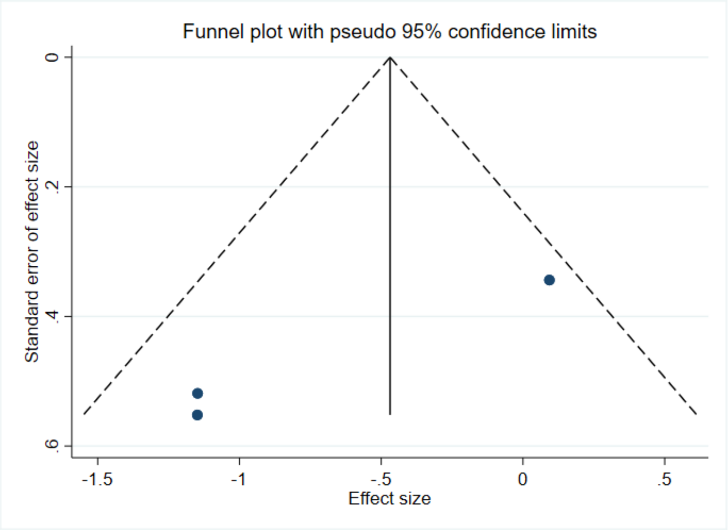

Figure S18. Funnel plot of min. ankle joint power

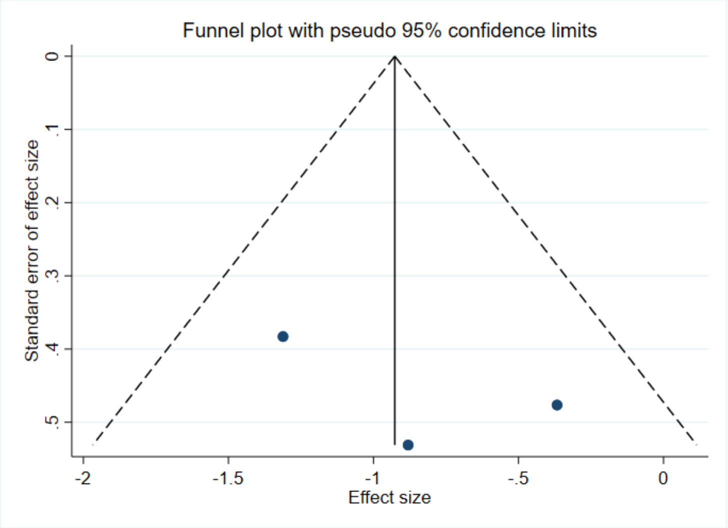

Figure S19. Funnel plot of max. ankle joint power

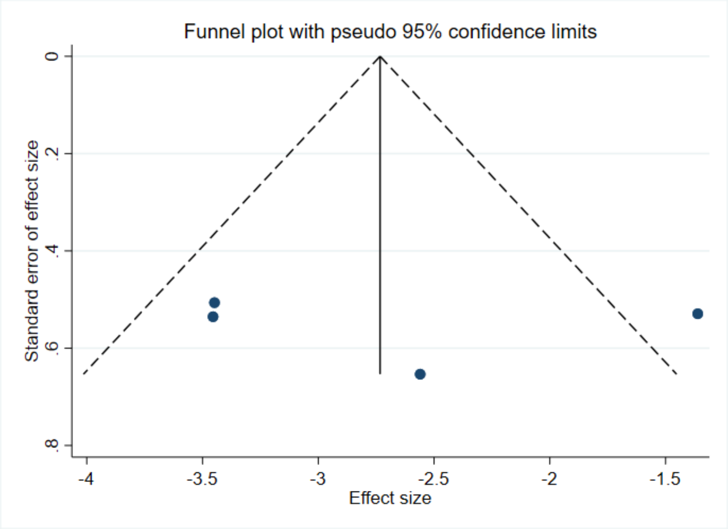

Figure S20. 60°/s isokinetic knee extension strength

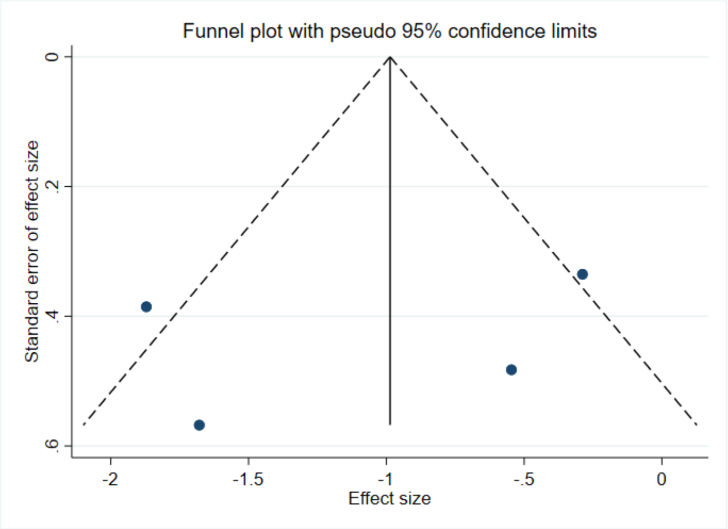

Figure S21. 60°/s isokinetic knee flexion strength

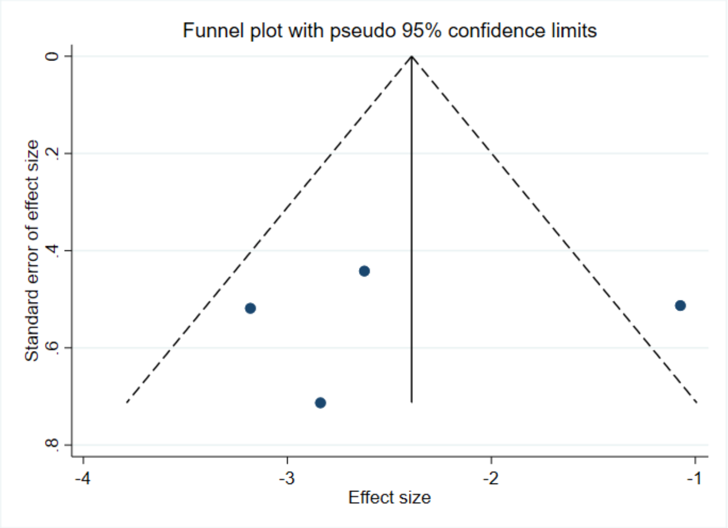

Figure S22. 180°/s isokinetic knee extension strength

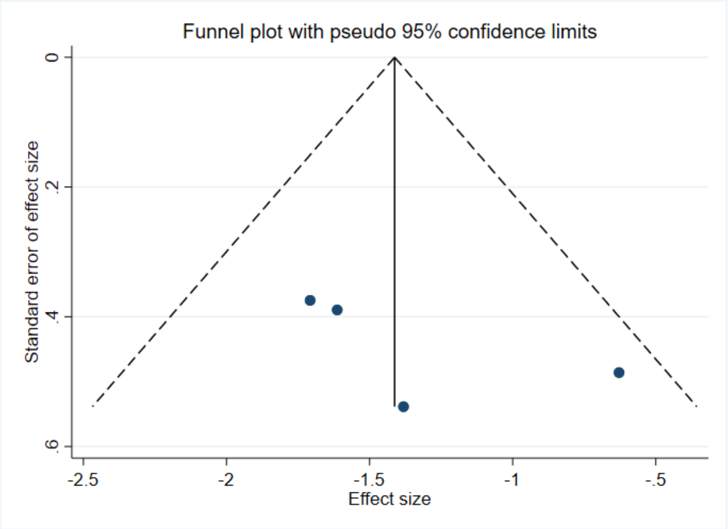

Figure S23. 180°/s isokinetic knee flexion strength
