# Supplementary material for: Lower Limb Biomechanical Outcomes Following Endoprosthetic Reconstruction for Distal Femur and Proximal Tibia Bone Tumors: A Systematic Review and Meta-Analysis
Source: Bioengineering (Basel). 2025 Nov 28;12(12):1310. doi: 10.3390/bioengineering12121310 (PMC12730080; doi:10.3390/bioengineering12121310)
Supplement: Supplementary file 1 [file bioengineering-12-01310-s001.zip › Supplementary Material-GRADE evidence profile.pdf]

GRADE evidence

Certainty assessment							№ of patients		Effect		Certainty	Importance
№ of studies	Study design	Risk of bias	Inconsistency	Indirectness	Imprecision	Other considerations	Intervention	Control	Relative (95% CI)	Absolute (95% CI)		
**Gait velocity (follow-up: mean 64.02 months)**												
10	non-randomised studies	not serious	not serious	not serious	not serious	dose response gradient	142	149	-	SMD **1.28 SD lower** (1.73 lower to 0.83 lower)	⊕⊕⊕◯ Moderate	CRITICAL
**Cadence (follow-up: mean 50.7 months)**												
7	non-randomised studies	not serious	not serious	not serious	not serious	dose response gradient	107	118	-	SMD **1.36 SD lower** (1.91 lower to 0.8 lower)	⊕⊕⊕◯ Moderate	CRITICAL
**Stride length (follow-up: mean 65.61 months)**												
5	non-randomised studies	not serious	not serious	not serious	not serious	none	86	104	-	SMD **0.63 SD lower** (1.1 lower to 0.15 lower)	⊕⊕◯◯ Low	IMPORTANT
**Step length (follow-up: mean 65.81 months)**												
5	non-randomised studies	not serious	not serious	not serious	not serious	none	67	99	-	SMD **0.6 SD lower** (0.93 lower to 0.27 lower)	⊕⊕◯◯ Low	IMPORTANT
**Duration of stance phase (follow-up: mean 82.79 months)**												
5	non-randomised studies	not serious	not serious	not serious	not serious	none	57	67	-	SMD **0.65 SD lower** (1.03 lower to 0.27 lower)	⊕⊕◯◯ Low	IMPORTANT
**Max. vertical force during early stance (follow-up: mean 61.24 months)**												
3	non-randomised studies	not serious	not serious	not serious	not serious	none	40	36	-	SMD **1.54 SD lower** (2.46 lower to 0.62 lower)	⊕⊕◯◯ Low	IMPORTANT
**Max. vertical force during late stance (follow-up: mean 61.24 months)**												

Certainty assessment							№ of patients		Effect		Certainty	Importance
№ of studies	Study design	Risk of bias	Inconsistency	Indirectness	Imprecision	Other considerations	Intervention	Control	Relative (95% CI)	Absolute (95% CI)		
3	non-randomised studies	not serious	not serious	not serious	not serious	none	40	36	-	SMD **1.34 SD lower** (1.86 lower to 0.82 lower)	⨁⨁◯◯ Low	IMPORTANT
**Peak hip flexion during early stance (follow-up: mean 99.04 months)**												
3	non-randomised studies	not serious	not serious	not serious	not serious	none	33	35	-	SMD **0.14 SD higher** (0.34 lower to 0.61 higher)	⨁⨁◯◯ Low	IMPORTANT
**Peak hip extension (follow-up: mean 99.04 months)**												
3	non-randomised studies	not serious	not serious	not serious	not serious	none	33	35	-	SMD **0.38 SD lower** (0.86 lower to 0.1 higher)	⨁⨁◯◯ Low	IMPORTANT
**Knee flexion at initial contact (follow-up: mean 61.24 months)**												
3	non-randomised studies	not serious	not serious	not serious	not serious	none	40	36	-	SMD **0.32 SD lower** (0.17 lower to 0.43 higher)	⨁⨁◯◯ Low	IMPORTANT
**Peak knee flexion during early stance (follow-up: mean 81.07 months)**												
4	non-randomised studies	not serious	serious[a]	not serious	not serious	publication bias strongly suspected[b]	49	45	-	SMD **1.55 SD lower** (2.48 lower to 0.63 lower)	⨁◯◯◯ Very low[a,b]	IMPORTANT
**Peak dorsiflexion during stance (follow-up: median 99.04 months)**												
3	non-randomised studies	not serious	serious[c]	not serious	not serious	none	33	35	-	SMD **0.29 SD higher** (1.72 lower to 2.3 higher)	⨁◯◯◯ Very low[c]	IMPORTANT
**Peak plantar flexion during stance (follow-up: median 99.04 months)**												
3	non-randomised studies	not serious	not serious	not serious	not serious	none	33	35	-	SMD **1.62 SD higher** (0.51 higher to 2.73 higher)	⨁⨁◯◯ Low	IMPORTANT

Certainty assessment							№ of patients		Effect		Certainty	Importance
№ of studies	Study design	Risk of bias	Inconsistency	Indirectness	Imprecision	Other considerations	Intervention	Control	Relative (95% CI)	Absolute (95% CI)		
**Max. knee extension moment during early (follow-up: mean 99.04 months)**												
3	non-randomised studies	not serious	not serious	not serious	not serious	publication bias strongly suspected strong association[d]	33	35	-	SMD **2.61 SD lower** (4.01 lower to 1.21 lower)	⊕⊕◯◯ Low[d]	IMPORTANT
**Max. plantarflexion moment (follow-up: mean 99.04 months)**												
3	non-randomised studies	not serious	not serious	not serious	not serious	strong association	33	35	-	SMD **1.68 SD lower** (2.25 lower to 1.11 lower)	⊕⊕⊕◯ Moderate	IMPORTANT
**Min. knee joint power during early stance (follow-up: mean 99.04 months)**												
3	non-randomised studies	not serious	not serious	not serious	not serious	none	33	35	-	SMD **1.52 SD higher** (0.96 higher to 2.07 higher)	⊕⊕◯◯ Low	IMPORTANT
**Max. knee joint power during early stance (follow-up: mean 99.04 months)**												
3	non-randomised studies	not serious	serious[e]	not serious	not serious	publication bias strongly suspected strong association[b]	33	35	-	SMD **2.62 SD lower** (4.05 lower to 1.19 lower)	⊕◯◯◯ Very low[b,e]	IMPORTANT
**Min. ankle joint power (follow-up: mean 99.04 months)**												
3	non-randomised studies	not serious	not serious	not serious	not serious	none	33	35	-	SMD **0.65 SD lower** (1.53 lower to 0.23 higher)	⊕⊕◯◯ Low	IMPORTANT
**Max. ankle joint power (follow-up: mean 99.04 months)**												
3	non-randomised studies	not serious	not serious	not serious	not serious	none	33	35	-	SMD **0.93 SD lower** (1.44 lower to 0.42 lower)	⊕⊕◯◯ Low	IMPORTANT
**60°/s isokinetic knee extension strength (follow-up: mean 59.46 months)**												

Certainty assessment							№ of patients		Effect		Certainty	Importance
№ of studies	Study design	Risk of bias	Inconsistency	Indirectness	Imprecision	Other considerations	Intervention	Control	Relative (95% CI)	Absolute (95% CI)		
4	non-randomised studies	not serious	serious[f]	not serious	not serious	very strong association	56	56	-	SMD **2.63 SD lower** (3.36 lower to 1.63 lower)	⊕⊕⊕◯ Moderate[f]	CRITICAL
**60°/s isokinetic knee flexion strength (follow-up: mean 59.46 months)**												
4	non-randomised studies	not serious	serious[g]	not serious	not serious	none	56	56	-	SMD **1.07 SD lower** (1.88 lower to 0.26 lower)	⊕◯◯◯ Very low[g]	CRITICAL
**180°/s isokinetic knee extension strength (follow-up: mean 59.46 months)**												
4	non-randomised studies	not serious	not serious	not serious	not serious	very strong association	56	56	-	SMD **2.41 SD lower** (3.34 lower to 1.47 lower)	⊕⊕⊕⊕ High	CRITICAL
**180°/s isokinetic knee flexion strength (follow-up: mean 59.46 months)**												
4	non-randomised studies	not serious	not serious	not serious	not serious	none	56	56	-	SMD **1.41 SD lower** (1.84 lower to 0.99 lower)	⊕⊕◯◯ Low	CRITICAL

CI: confidence interval; **SMD:** standardised mean difference

Explanations

a. Moderate heterogeneity observed (I^2=70.04%) across the studies.
b. This was confirmed by the statistically significant Egger's test ($P < 0.05$) and clear asymmetry observed in the funnel plot. However, a crucial sensitivity analysis using the trim-and-fill method was performed to adjust for this potential bias. The results showed that the adjusted pooled estimate was consistent with the original result, indicating that while publication bias is likely present, it does not appear to have fundamentally altered the direction of our primary findings.
c. Moderate heterogeneity observed (I^2=91.77%) across the studies.
d. Moderate heterogeneity observed (I^2=70.85%) across the studies.
e. Moderate heterogeneity observed (I^2=71.54%) across the studies.
f. Moderate heterogeneity observed (I^2=70.43%) across the studies.
g. Moderate heterogeneity observed (I^2=72.70%) across the studies.
